# Ambient Parameter Monitoring in Fresh Fruit and Vegetable Supply Chains Using Internet of Things-Enabled Sensor and Communication Technology

**DOI:** 10.3390/foods11121777

**Published:** 2022-06-16

**Authors:** Anna Lamberty, Judith Kreyenschmidt

**Affiliations:** 1 Department of Fresh Produce Logistics, Hochschule Geisenheim University, 65366 Geisenheim, Germany; judith.kreyenschmidt@hs-gm.de; 2 Projects and Innovation Department, Euro Pool System International (Deutschland) GmbH, 53332 Bornheim, Germany

**Keywords:** wireless sensing, Internet of things, cold chain management, fruit and vegetables, postharvest quality, shelf life, food waste

## Abstract

Up to half of the global fruit and vegetable production is wasted or lost along the supply chain, causing wastage of resources and economic losses. Ambient parameters strongly influence quality and shelf life of fresh fruit and vegetables. Monitoring these parameters by using Internet of things (IoT)-enabled sensor and communication technology in supply chains can help to optimize product qualities and hence reduce product rejections and losses. Various corresponding technical solutions are available, but the diverse characteristics of fresh plant-based produce impede establishing valuable applications. Therefore, the aim of this review is to give an overview of IoT-enabled sensor and communication technology in relation to the specific quality and spoilage characteristics of fresh fruit and vegetables. Temperature, relative humidity (RH), O_2_, CO_2_ and vibration/shock are ambient parameters that provide most added value regarding product quality optimization, and can be monitored by current IoT-enabled sensor technology. Several wireless communication technologies are available for real-time data exchange and subsequent data processing and usage. Although many studies investigate the general possibility of monitoring systems using IoT-enabled technology, large-scale implementation in fresh fruit and vegetable supply chains is still hindered by unsolved challenges.

## 1. Introduction

It is estimated that 40–50% of the global fruit and vegetable production is lost or wasted along the supply chain, which is approximately half of the total amount of food waste [1,2]. This leads not only to wastage of resources and corresponding avoidable greenhouse gas emissions, but also creates huge economic losses [1,2]. Therefore, reducing the amount of food wasted and lost along the supply chain is of major importance regarding sustainability and economical aspects [1].

There is no single reason for the high losses in the fresh fruit and vegetable sector, but several characteristics make these products particularly prone to wastage. They are highly perishable with shelf life and postharvest quality being strongly influenced by the environmental conditions during storage and transportation [3]. Furthermore, the seasonality of many fruits and vegetables can lead to discrepancies between the availability and the market situation, causing product losses [4]. An additional aspect is the increasing consumer demand for the highest product quality [3]. Together with rising quality standards enforced by retail, this results in high losses of less aesthetically perfect products, especially when marketing alternatives are lacking [1,4]. 

Both the postharvest quality retention and the shelf life of fresh fruit and vegetables are influenced by a range of ambient parameters such as temperature, relative humidity (RH), gas atmosphere and shock or vibration [5,6]. Monitoring and controlling these parameters during storage and distribution contributes to optimized product qualities and reduction of losses in the respective supply chains [5]. Combining the product’s temperature history with shelf life models allows predictive shelf life calculations and improved logistic decisions [7,8]. 

A common way for ambient parameter tracking is using respective sensor technology. Various solutions have been developed in recent years [6]. With the progress of the IoT, monitoring systems with wireless real-time data exchange between numerous system components become possible [9,10]. This is a huge advantage over most conventional systems such as dataloggers, graphical temperature recorders, time-temperature integrators and smart labels, which require physical interaction with the device for data extraction [11]. Luo et al. [12] proposed an IoT-based monitoring system, including layers for sensing (sensing layer), transmitting and processing (network layer) and management of data (application layer). The corresponding technologies are emerging and partly already commercially available.

However, despite the rapid technological development and the potential for reducing food loss and waste, the implementation of IoT-based monitoring systems is still facing unsolved challenges. Ambient parameter tracking using IoT technologies has been investigated in various studies [13,14,15], but the corresponding detailed concepts on data processing and cross-chain implementation are missing. Fruit and vegetables are a complex product group with various freshness and quality characteristics. Depending on the product and its spoilage kinetics, different ambient parameters are important and useful to measure. This leads to a broad range of monitoring requirements for fruit and vegetables on the one hand. On the other hand, a variety of IoT-based technologies is available, but there are currently no generic guidelines that help actors to decide which technology fits best for their specific supply chains. 

The central objective of this review is to give an overview of IoT-enabled sensor and communication technology for monitoring ambient parameters in relation to the specific quality and spoilage characteristics of fresh fruit and vegetables. Current technological solutions were reviewed for their potential regarding useful applications in supply chains of fresh plant-based produce. This enables actors to make a more informed choice about which technology could be valuable for specific supply chains, contributing to optimized product qualities and reduction of losses.

For bibliographic retrieval, scientific databases (Web of Science, Google Scholar, Wiley) were used to collect scientific literature. The research was conducted on the main topics of spoilage characteristics, quality-relevant ambient parameters, IoT sensor and communication technology, and application studies. Examples of keywords used for the search are “fruit and vegetables” and “shelf life”, “wireless sensing” and “postharvest quality”, “IoT” and “food supply chain”, “IoT” and “cold chain management”. For examples of sensor technology and related technical information, sources directly retrieved from the web were used additionally, since scientific literature is not exhaustive enough for this kind of information. After reviewing the search results, 105 bibliographic references from scientific databases and 22 web sources were included in this review. 

Firstly, spoilage mechanisms of fresh fruit and vegetables and their implications for the quality and shelf life are described in Section 2. This is followed by Section 3, in which relevant ambient parameters and the corresponding IoT-enabled sensors are presented. In Section 4, IoT communication technologies enabling wireless data exchange are described. In the discussion in Section 5, the findings from the previous sections are integrated. The potential, but also the challenges of ambient parameter monitoring in fresh fruit and vegetable supply chains using IoT-based sensor and communication technology are pointed out. 

## 2. Spoilage Characteristics and Shelf Life of Fresh Fruit and Vegetables

The term “fruit and vegetables” covers a broad range of products originating from different plant parts, including tissue types such as leaves, reproductive organs and other organs such as roots [9,16]. The physiological variety of fruit and vegetables leads to diverse characteristics regarding quality and spoilage. Some products such as berries and lettuce are highly perishable with only a few days of shelf life, although others such as apples, pears and onions can be stored for months. Accordingly, the requirements for postharvest quality retention vary. Depending on each product and its specific spoilage characteristics, different ambient parameters are relevant and thus must be monitored to enable quality optimization and dynamic shelf life calculations [5,7,17]. However, common spoilage mechanisms that are influenced by certain ambient parameters can be observed. Categorizations after those mechanisms can help to establish which products can be transported or stored together without accelerated quality decline [18]. 

### 2.1. Respiration

Postharvest fruit and vegetables are living tissue with continuing respiration that leads to further ripening and eventually over-ripeness and senescence. Respiration describes the process of metabolizing O_2_ from the air together with organic molecules (e.g., sugar) from the tissue to intermediate compounds and eventually CO_2_ and water [19,20]. Many metabolic processes that have direct influence on quality attributes such as firmness, sugar content and aroma are depended on respiration [19]. If these quality parameters deteriorate beyond a certain acceptance threshold, the product is spoiled. Therefore, respiration rate and shelf life are mostly inversely correlated, meaning that a higher respiration rate implies a shorter shelf life [19]. A comprehensive table with respiration rates for fruit and vegetables can be found in Gross et al. [21]. Some authors classify the respiration rates relatively to each other to indicate whether a product has a “high” or “low” respiration rate and accordingly a longer or shorter shelf life [5,16,19]. Respiration is strongly influenced by the ambient temperature and the gas atmosphere surrounding the product [20]. Lowering respiration rates via temperature control and/or modified gas atmosphere (e.g., reduction of oxygen concentration) can lead to an extended storage life for some commodities [16,22].

A special type of respiratory behavior can be observed in climacteric fruits during ripening. This also includes some products which are commonly termed as “vegetables” but are physiologically fruit-vegetables, such as tomatoes. For non-climacteric commodities hardly any changes are observed in their respiration pattern, climacteric fruits show a sudden strong increase in respiration rate to a maximum (climacteric peak) that is followed by a rapid decline [5]. Examples of climacteric fruits are apples, bananas, mangoes, melons, pears and tomatoes. Climacteric behavior can be advantageous if products are transported over long distances, such as overseas transport, because it allows for continued ripening after harvest. It is common practice that climacteric fruits are harvested mature but preclimacteric and are ripened under controlled conditions at their destination [9]. The gaseous plant hormone ethylene strongly influences the onset and progression of climacteric ripening [23]. Therefore, optimized conditions regarding temperature and ethylene concentration in the surrounding atmosphere are necessary for preventing product loss by enhanced respiration or sudden climacteric ripening.

### 2.2. Transpiration

Transpiration describes the physiological process in which water vapor evaporates from the product’s surface to the surrounding air. It continues after harvest and causes progressing water loss in the product, resulting in turgor loss, wilting, shriveling and shrinking [24]. This leads to a deterioration of quality parameters such as taste, turgidity or texture, until the product is unacceptable to the consumer [5,9,25]. The degree of water loss that is still accepted is product-specific [5,16]. Next to visible quality parameters, the nutritional value can also decrease from water loss [16]. Quality decline and spoilage due to moisture loss concerns primarily leafy vegetables, vegetables with leaves on top, citrus fruit and products with a high surface area to fresh weight ratio, such as bell peppers [16,24]. The ambient RH and temperature play a crucial role in reducing postharvest transpiration [5,9]. High RH (95–100%) for optimum shelf life and quality retention during storage and distribution is recommended for many products [21]. Only few products, such as onions and garlic, require a lower RH [18].

### 2.3. Microbial Growth

All fruits and vegetables are nutrient-rich and high in water activity, providing a suitable habitat for various microorganisms. The main spoilage flora is composed of saprophytic microorganisms derived from the surroundings in the field or orchard (e.g., soil, air, insects, irrigation water, hands of personnel) [26]. Microbial spoilage can occur for any product and often is the limiting factor for its shelf life, because of altered quality parameters such as smell, flavour, firmness, texture, and colour. For vegetables, bacterial spoilage is common [27]. Organisms such as Erwinia carotovora and Pseudomonas spp. frequently cause spoilage in onions, crucifers, peas, beans, carrots, potatoes, asparagus, celery, and lettuce [27]. Due to their acidity, fruit are more likely to be infected by fungi [27]. Berries in particular are very susceptible to fungal infections and subsequent spoilage. Main spoilage organisms are Rhizopus stolonifer and Botrytis cinerea, both leading to high market losses [26]. Likewise, citrus fruit are frequently spoiled by fungi, especially by the species Penicillium italicum and *Penicillium digitatum* [26]. In addition, fruit and vegetables can be associated with pathogens naturally occurring in the plant environment or by contamination via various sources. Recent outbreaks in Europe have for instance been reported for Norovirus in radish sprouts and *Salmonella enteritidis* in cucumbers and tomatoes [28,29]. Growth of microorganisms is strongly dependent on ambient parameters such as temperature, RH, and gas atmosphere [30]. Therefore, close control and monitoring of these parameters is essential for delaying microbial spoilage and preventing the growth of pathogens. 

### 2.4. Degradation of Internal Components

Metabolic processes such as respiration and enzymatic activity continuously alter the internal product composition. Degradation of components can be visible, as in the loss of green colour in broccoli heads, because of chlorophyll breakdown [16,31], but this is not always necessarily so. Although the loss of components is not always related to spoilage, it is an important quality aspect. Beneficial health effects are associated with compounds such as vitamins, carotenoids, flavonoids, phytosterols and phenols [30]. These might be reduced or lost if products are stored under improper conditions. Several studies show that the ambient temperature and RH have an influence on the degradation of nutrients such as ascorbic acid, chlorophyll, carotenoids, lipids and glucosinolates in different fruit and vegetables [5,32,33,34,35]. Bergquist et al. [36] state that the ascorbic acid content in baby spinach is so closely correlated with the ambient conditions that it could be used as a shelf life indicator. Therefore, control of ambient parameters such as temperature and RH also has a direct influence on the nutritional quality of a product. 

### 2.5. Damage and Injury

Fresh fruit and vegetables are delicate products and easily damaged by shocks or vibration during handling, storage, and distribution. Mechanical injuries and bruises promote the beforementioned deterioration mechanisms strongly, leading to faster quality decline and spoilage. They increase respiration, transpiration, ethylene production and accelerate enzymatic activity [5,22,26]. Furthermore, mechanical damage results in contact areas for microorganisms, especially for moulds, and leakage of cellular fluid that provides good conditions for growth of bacteria [21,26]. A special type of injury is the so-called chilling injury. Commodities that are susceptible to this condition can suffer from different physiological disorders when exposed to low temperatures for a certain time [37]. For example, apples, pomegranates, and pineapples can develop internal browning, and pitting can occur in oranges, papayas, watermelons, and cucumbers [37]. The threshold temperature is product-specific, therefore chilling injury cannot only be prevented by close temperature control, but also by knowing the optimum conditions for each commodity. 

## 3. Ambient Parameter Monitoring and Sensor Technology 

From the spoilage mechanisms discussed before, it becomes obvious that ambient parameters strongly influence the quality and shelf life of fruit and vegetables. Purposeful and continuous monitoring is required to provide safe, fresh and nutritious products. Traditional ambient parameter tracking in fruit and vegetable supply chains relies on non-connected, partly even non-digitalized practices [3]. New approaches are under investigation to complement or replace those monitoring methods. The lack of real-time information is a particular problem that new solutions try to overcome to improve food safety and reduce waste [38,39]. Moreover, the availability of real-time information on ambient conditions is a critical requirement for predictive shelf life calculations [7,40]. One approach to provide this information is to use IoT-based sensors with wireless communication technologies. 

### 3.1. Temperature

Temperature is the most important ambient parameter in the context of quality retention and shelf life because it strongly influences all spoilage mechanisms of fresh fruit and vegetables as discussed in Section 2. For instance, Nunes and Emond [41] found that the shelf life of raspberries was reduced by 50% if the storage temperature was 10 °C instead of 0 °C. For blueberries, a shelf life shorter by 3 days was observed for storage at 10 °C compared to storage at 0 °C [42]. Also for arugula, increasing the storage temperature from 0 °C to 7 °C reduces the shelf life by several days, depending on the season and the harvest batch [43]. For products that are sensitive to chilling injury, such as mangoes, avocados, bananas, papayas and pineapples, not only a temperature increase, but also a lower than optimum temperature can negatively impact their quality. Islam et al. [44] reported first symptoms of chilling injury in mango after 10 days of storage at 5 °C, whereas at 10 °C the onset of symptoms only started after 20 days. Another study on papayas showed that chilling injury symptoms were much more severe in fruit stored at 5 °C for 7 days than in fruit stored at 10 °C for 7 or even 14 days [45]. These examples illustrate that particularly for very perishable products that require strict refrigeration and for chilling sensitive products, real-time temperature monitoring could be beneficial to prevent accelerated quality losses. Technologically, this is already possible. Temperature sensors are widely available and account for up to 80% of the global sensor market [46]. Although traditionally thermocouples were common, in most IoT temperature sensors resistive temperature devices or thermistors are used [6]. Both types are inexpensive and measure the temperature accurately over a wide range [6]. Given the availability of cost-efficient IoT temperature devices, an application for optimized product quality and shelf life can be profitable for all fruit and vegetables.

### 3.2. Relative Humidity

During storage and distribution, it is essential to find the right level of RH that minimizes moisture loss but at the same time prevents microbial growth [5]. For example, Mahajan et al. [47] observed increasing weight loss in fresh mushrooms with decreasing RH. They state that the RH has the largest effect on lowering the transpiration rate, but that a high RH at the same time can promote growth of certain microorganisms [47]. Therefore, real-time monitoring and control of RH is especially important for fresh fruit and vegetables that are either prone to water loss or very susceptible to microbial decay, such as berries and citrus fruit. Apart from moisture loss, RH can adversely influence further quality characteristics. Jones et al. [35] describe that broccoli heads lose visual quality and glucosinolates’ content faster at low RH than at high RH when stored at ambient temperature. For carrots, studies indicate that high RH is beneficial for maintaining levels of ß-carotene, ascorbic acid, glucose and fructose [48]. This shows that monitoring of RH with IoT technology can be beneficial for several fruit and vegetable supply chains. Different technologies are possible, including optical, gravimetric, capacitive, resistive, piezo-resistive and magnetoelastic sensors [49]. For application in IoT devices, mostly capacitive and resistive sensors are used, because of their robustness, small size and low power consumption [6]. IoT solutions for measuring RH are widely available and often combined with temperature sensors [6].

### 3.3. Ethylene

The gaseous plant hormone ethylene enhances metabolic processes in many fruit and vegetables, but especially stimulates ripening in climacteric fruits [23]. Already extremely low concentrations can affect sensitive products [23,50]. Detrimental effects include accelerated senescence [23], loss of chlorophyll and yellowing in green-colored vegetables [16,22,23], accumulation of bitter compounds in carrots and parsnips [16,22,51] and excessive flesh softening, e.g., in kiwifruit and watermelons [51,52]. Unfavorable conditions can arise when multiple commodities are stored together, since the presence of ethylene producing products can lead to quality decline in sensitive ones [18]. Even when ethylene is intentionally added for controlled ripening and de-greening purposes, the concentrations should be monitored and controlled carefully [53]. Therefore, real-time monitoring of ethylene concentrations would be highly valuable during transport and storage of climacteric fruits and products that are sensitive to exposure. Several technological approaches exist; however, challenges regarding specificity, selectivity, price, size and stability in a harsh measurement environment remain [50,51]. Until now, ethylene sensors with sufficient accuracy are too costly for an application in IoT devices, but promising progress is being made, particularly in the field of chemiresistors, chemicapacitors and NDIR spectroscopy [6,50]. Another possibility for further cost reduction could be the development of “threshold” sensors that only transmit data if the ethylene concentration reaches a certain level, dependent on the respective product [50].

### 3.4. Oxygen and Carbon Dioxide

In controlled atmosphere (CA) storage, altered oxygen and carbon dioxide concentrations are used to reduce respiration, ethylene production and enzymatic activity, which lead to better nutrient retention and slower microbial growth [5,54]. For example, Schouten et al. [31] found that broccoli heads stored in 1.5 kPa O_2_/15 kPA CO_2_ at 18 °C were still green after 10 days whereas broccoli heads in other CA storage conditions turned yellow. Chung et al. [55] showed that for apples of the variety “Fuji”, the flesh firmness was retained significantly better under CA conditions than in air storage. Comparable beneficial effects are observed for many commodities, but only for some the beneficial effects are large enough for a profitable commercial use [22]. CA storage is commonly applied for apples, pears, cabbages and, to a lesser extent, to bananas, onions, kiwifruit, avocados, kakis, strawberries during long-distance transport [52,54]. Injuries and physical disorders can occur when the concentrations shift outside the suitable range [54]. Therefore, real-time monitoring of oxygen or carbon dioxide concentrations can be valuable when using CA. For both gasses, several sensing technologies are established, but not all of them are suitable for an IoT application [56]. Finner and Zomer [6] propose the use of electrochemical or fluorescence sensors for oxygen detection and NDIR sensors or metal oxides sensors for carbon dioxide detection because they are inexpensive and sufficiently accurate [6].

### 3.5. Shock and Vibration

During transport and distribution, products are loaded and unloaded several times, and may experience shock or vibration. Fresh fruit and vegetables are easily damaged during those incidents. This is relevant for quality and shelf life because mechanical damage or injuries strongly promote quality loss and spoilage. For instance, Martinez-Romero et al. [57] measured significantly higher respiration rates during storage for damaged apricots than for non-damaged ones. For carrots, different processing steps from mechanical harvesting to packing can cause a huge amount of loss through damage [25]. The severity of the damage also plays an important role for quality retention and shelf life, as Ariffin et al. [58] show for different spinach types. Hence, using accelerometers (motion sensors) for detecting shock or vibration can be useful for very sensitive products such as berries, but also for many other commodities. Most suitable accelerometers for IoT devices are based on microelectrochemical systems (MEMS), because they are small, inexpensive and accurate [59,60]. These devices are widely available and can be used for detecting shock or vibration throughout the chain by simple attachment to a pallet [60].

Table 1 gives an overview of the presented ambient parameters and the corresponding sensor technology suitable for an IoT application. In addition, fresh fruit and vegetables are listed for which monitoring of the respective ambient parameter could bring value by preventing product losses through unexpected deterioration. The examples for commercially available sensors contain ready-to-use solutions including battery, communication modules, etc. For ethylene, only sensing units without inbuilt battery or connectivity were found. Depending on the intended application it can also be favorable to develop a customized sensor from single modules. 

## 4. Wireless Communication Technologies for IoT-Enabled Data Transmission

A key advantage of ambient parameter monitoring with IoT technology is that the gathered data can be transmitted and processed in real-time, so that actions can be taken accordingly [78]. In the context of IoT architecture, this is done via the network layer [10,12]. It transmits data that is collected by the device layer (which includes sensors as described in the previous section) using wired or wireless communication technologies [10]. This review focuses on wireless communication technologies such as Bluetooth, RFID or cellular communication, because they have multiple advantages over wired ones regarding cost, size and flexibility [79]. The technologies mentioned here are described with a focus on potential applications in fresh fruit and vegetable supply chains. More extensive information can be found in Ruiz-Garcia et al. [79], Mekki et al. [80], Feng et al. [81] and Cao et al. [82].

### 4.1. Near-Field Communication, Radio Frequency Identification, Zigbee and Bluetooth

The technologies Near-Field Communication (NFC), Radio Frequency Identification (RFID), Zigbee and Bluetooth operate in short range data transmission with transmission distances from a few centimeters up to a few hundred meters and use unlicensed frequency bands [79,80,81,83]. NFC uses the frequency band of 13.56 MHz and provides the shortest transmission range with up to 10 cm [83]. Important advantages of this technology are its low costs, international standardization and easy integration into existing processes because NFC tags are readable with almost every smartphone [82]. An interesting aspect is the possibility of using energy harvesting to power passive NFC tags, making them independent from a battery and more sustainable [82]. Zigbee and Bluetooth enable data transmission over a range of up to 100 m [81,82]. Both are technologies with relatively low costs and low power consumption [81]. On the one hand, Zigbee can be preferred over Bluetooth when a large number of sensor devices should be connected, or a flexible network structure is needed [84]. On the other hand, Bluetooth can outperform Zigbee regarding energy efficiency, especially when the newest Bluetooth Low-Energy (BLE) standard is used [83]. Low-cost and low-power communication are also characteristics of RFID, when passive or semi-passive tags are used. These tags send their data only by reflecting or modulating the signal coming from the reader, although active tags have their own battery which enables a stronger signal and a larger range [79]. For passive and semi-passive tags, the data transmission range is limited to 10 m, and for active tags, up to 100 m is possible [85]. In food supply chains, RFID applications were originally intended to replace optical barcodes for traceability purposes [79,86]. However, interest has grown in further applications of RFID tags because they are robust, able to work under harsh conditions and quickly installed [87]. If sensors are integrated in RFID tags, they can enable automatic identification of goods, e.g., a pallet with fruit, combined with the ambient parameter history profile of that pallet [87]. Drawbacks of RFID are readability problems when surrounded by metal or products with high water content, and limited real-time data collection during transport [85,87]. Furthermore, the lack of standardized global frequency use hinders international RFID applications [85]. These are major disadvantages for applications in fresh fruit and vegetable supply chains, which often operate internationally. The short data transmission range of RFID, but also of NFC, Bluetooth and Zigbee is an additional limiting factor that obstructs many IoT applications [80]. Therefore, various authors investigated the combined use of short range and other wireless communication technologies to overcome the disadvantage of short transmission distance. Some examples are presented at the end of this section.

### 4.2. Wi-Fi and Cellular Communication

The transmission distance of Wi-Fi is up to 100 m, which is comparable to Zigbee [81,83]. Costs are relatively low because Wi-Fi uses an unlicensed band, but this can also lead to interference with other devices sharing the same band [88,89]. Therefore, the Quality of Service is not guaranteed for this technology [88]. However, it enables a faster data transmission speed than other short range communication technologies and many facilities along a supply chain already have a Wi-Fi connection [89]. The high data rate and therefore also higher power consumption can be unfavorable for direct connection to sensor devices that depend on battery power. The same is applicable for cellular communication technologies such as 2G, 3G, 4G and 5G which enable a high data rate and thus have a high power consumption [81]. IoT-enabled sensors for ambient parameter monitoring require only a low data rate, shifting the preferences towards a communication technology that is more energy efficient [80]. Nevertheless, applications that transmit larger amounts of data can benefit from cellular communication because they are not duty cycle limited [90]. An advantage of cellular networks is their large coverage for long distance data transmission of several kilometers due to an already existing infrastructure [80,90]. Moreover, modern specifications such as 5G offer a very high Quality of Service and the possibility of connecting a massive number of devices [88]. 

### 4.3. Low Power Wide Area Networks

An emerging communication technology for use in an IoT context are low power wide area networks (LPWAN). LPWAN technologies provide low cost, low power, low data rate, large coverage and long distance data transmission connections [81,91]. Technologies that are often used are for example LoRa and narrowband (NB)-IoT. A detailed comparison study is provided by Mekki et al. [80]. An important aspect is that LoRa uses an unlicensed frequency band, and NB-IoT uses a licensed one. This results in a guaranteed Quality of Service for NB-IoT because no duty cycle limits hinder the data throughput [80,90]. Key advantages of LoRa are its lower costs for connecting end devices and no operating costs for data transmission [80,91]. Additional smaller differences in for example the maximum number of end devices, payload length and latency might be important to consider when choosing the right technology for a certain application. For real-time parameter monitoring in fresh fruit and vegetable supply chains, the coverage in suburban and rural areas can be a critical factor. Although LoRa and NB-IoT are expected to further expand their coverage, this aspect is important especially for internationally operating supply chains. 

All communication technologies presented in this section have their specific strengths and weaknesses. Generally, the biggest differences are in the range of data transmission, the data rate, the power consumption, the standardization, the costs and the availability of guaranteed Quality of Service. It is important to distinguish between technologies that use licensed and unlicensed frequency bands for data transmission, because this has direct influence on the costs and possible interference problems. Wireless communication is a crucial part of IoT applications and therefore technologies will further develop (e.g., transformation to 6G) and new technologies will emerge. Unfortunately, interoperability is limited and establishing corresponding standards remains a challenge [83,91]. Additionally, various privacy concerns hinder actors from adopting these technologies on a large scale [3,39,78,92].

Regarding IoT-based ambient parameter monitoring in fresh fruit and vegetable supply chains, there are many approaches to overcome disadvantages of certain communication technologies by combining two or more of them. Only some of the studies proposing an IoT-based monitoring system also apply it in practice. Luo et al. [12] proposed a temperature and RH tracking system that combines RFID, Zigbee and cellular communication technologies, but did not test it in a real supply chain. Musa and Vidyasankar [93] described a similar approach using RFID and cellular networks for transmission of temperature, RH, carbon dioxide concentration and light intensity data in a blackberry supply chain. However, no validation was carried out in a real environment. Ou et al. [94] developed a system using NFC connection to a smartphone for monitoring temperature and RH during transport, but also no testing scenario was described. An example of an application in a real supply chain provided by Badia-Melis et al. [13] used RFID tags and a wireless sensor network based on Zigbee to monitor temperature and RH in commercial fruit storage warehouses. Data transmission was possible even across isolating doors [13]. More than 98% of the data packages were transmitted successfully, but they concluded that the large amount of RFID tags used was only practical in a testing scenario [13]. 

A study by Ruiz-Garcia et al. [95] investigated the application of a Zigbee-based monitoring system inside a truck. It was observed that some sensor motes were not able to transmit any data due to absorption of the signal, depending on their position inside the truck. Regarding temperature measurements, they found that, on average, the temperature was outside the recommended level for 98% of the time [95]. 

Another application can be found in Zou et al. [96], who proposed a combination of RFID and Wi-Fi or cellular networks and used it in a supply chain for sweet melons from Brazil to Sweden to monitor temperature, RH, oxygen concentration, carbon dioxide concentration, and vibration or shock. They found that the dense packing of products inside the container lengthened the cooling time until the desired temperature was reached [96]. Furthermore, they measured critical CO_2_ concentrations at different handling steps, which could have a negative effect on product quality [96]. 

Zhu et al. [97] developed and evaluated a monitoring system that uses RFID and cellular communication for tracking temperature, RH, vibration, O_2_, CO_2,_ and ethylene in a fresh garlic scape supply chain. They were able to monitor the variability of these parameters during different supply chain steps [97]. Moreover, they state that the system enhanced the performance of the quality-relevant ambient parameters, resulting in a quality loss decrease from 20–30% to 15% [97]. The market price per kg of product increased by more than double. Still they discovered inefficiencies in their monitoring system that could be improved, for example regarding data quality, energy costs or adaptability to supply chain processes [97].

Some more examples of application studies are summarized in Table 2, together with an overview of the most important characteristics of the communication technologies as described above.

## 5. Discussion

The rapid development in the field of IoT technologies facilitates wireless real-time ambient parameter monitoring in fresh fruit and vegetable supply chains. This creates possibilities for reducing the amount of products lost and wasted along the chain. Firstly, an IoT-based monitoring system can be used for getting insight into the current product status, performing weakness analysis on parameter profiles, and enhancing process control. Secondly, the combination of a monitoring system with predictive food models enables prediction of the remaining shelf life in each step of the chain on actual sensor data [112]. This approach facilitates optimization of storage and logistics [17,78,113]. For example, instead of the conventionally used FIFO (first in first out) principle, the FEFO (first expired first out) principle could be applied in warehouse management, which allows for shelf life-driven distribution of products [17]. To realize this, shelf life models for respective products are required [112,114]. If fresh fruit and vegetable supply chains should benefit from the combined use of IoT-based sensors and dynamic shelf life, a range of specific and accurate models is needed.

This is a challenging task, since fresh fruit and vegetables are a complex product group with various quality and spoilage characteristics. It strongly depends on the product which ambient parameters have most influence on the quality and hence should be monitored and controlled for achieving added value. Nevertheless, certain commonalities can be identified. Quality loss and spoilage in fruit and vegetables are mainly driven by respiration, transpiration, microbial decay, degradation of components and mechanical damage or injury. Categorizations of products with similar storage requirements or ethylene sensitivity are developed to facilitate multi-commodity transport and storage [18,115,116]. Clustering approaches can deliver an important contribution to establish which ambient parameters are most important to monitor for which products. 

The complexity in choosing the relevant ambient parameters for each product is further increased by a vast range of options for IoT technologies. Regarding the device layer, sensor development is advanced, especially for monitoring of temperature and RH. Ready-to-use devices or modules for prototype customization are cost-efficient and commercially available on large scale. For the network layer, a variety of communication technologies is available that offer fast and simple data transmission. Most important characteristics include the data transmission range, the use of licensed or unlicensed spectrum, the power consumption, the costs and the standardization. Selecting the right combination of technologies is crucial for achieving added value with an IoT-based monitoring system. It is disadvantageous that there are currently no guidelines for supply chain actors who want to implement such a system on how to decide on appropriate technologies for their specific use.

Additionally, other factors besides product-related ones influence the choices of whether and which sensor and communication technology should be used. Particularly supply chain- and process-related aspects are important: the mode of transport affects the type and number of process steps involved and consequently the ambient conditions the product is exposed to. Land transport is most used for fruit and vegetables with distances up to thousands of kilometers [39]. Maintaining optimal conditions, especially temperature, is difficult during such long transportation. Studies show that the temperature conditions can vary significantly within a truck and even within a pallet, depending, among others, on the age and design of the transport/storage unit or the packaging of the products [3,117,118,119]. Same applies for air transport, which involves numerous handling steps that make the cold chain vulnerable to interruptions [39]. For products being exported to remote markets, sea transport in reefer containers is a standard part of the supply chain [120]. Interruption of the cold chain can occur frequently during container handling at the port terminals [120]. Additionally, real-time parameter monitoring during sea transport can be difficult because overseas connectivity is often based on expensive satellite communication [6]. These aspects lead to the problem that process-related requirements of implementing IoT sensor technology need to be established newly for every specific supply chain. This includes, for example, requirements regarding the placement of sensors, the minimum number of sensors and the measurement frequency. 

The complexity of international, multi-actor supply chains of fresh fruit and vegetables poses further challenges. For highest benefits regarding quality retention and reduction of product losses, a monitoring system should not only cover part of the supply chain but should be implemented cross-chain. Until now, operators in the supply chain are often reluctant to share corresponding data along the chain due to various privacy concerns [3,39,79,92]. However, information exchange is a basic requirement to successfully implement new quality-oriented concepts. Increased awareness among the actors regarding the (also monetary) advantages of a cross-chain monitoring system could improve this [39], as well as further information about the use of modern sensor and communication technology. It is important to understand which requirements different supply chain actors pose on an IoT-based monitoring system and the corresponding data platform, e.g., regarding data exchange, data storage, applications and interfaces. 

Even if actors are willing to share the information, simple data exchange along the chain is not always possible. Standards regarding data formats, data exchange and technical equipment are missing or mismatching [78,91,120,121]. Providers have generally been more interested in selling their proprietary solution than in investing in open standards [91,117]. Compatibility and integration of the multiple heterogenic devices and communication technologies are a big challenge for IoT-based systems [10]. This hinders efficient information exchange and data processing for quality and shelf life-related applications. The rise of blockchain-based solutions integrated into agri-food supply chains has potential to mitigate those aspects. Increased supply chain vulnerability and resulting security concerns of actors can be addressed by the possibility of immutable data exchange and privacy preservation [122,123,124]. Regarding interoperability, blockchain-based solutions can facilitate automated information sharing between actors because all actors are required to use the same data structure or standard [122,125]. Furthermore, combining IoT technologies with blockchain includes improved traceability, enhanced collaboration between actors, operational efficiencies and integration between systems of different actors [122,123,125]. 

Regarding monetary aspects, the costs and benefits of an IoT-based real-time monitoring system can be distributed unequally along the chain. Jedermann et al. [117] describe that the actor paying for installation and hardware costs is not necessarily the one who benefits most from the system. This can be seen as critical if missing concepts for sharing the costs and benefits along the supply chains might enhance already existing power asymmetries between actors. 

All these factors illustrate that the application of IoT-based sensor technology in fresh fruit and vegetables’ supply chains is influenced by many aspects and faces specific challenges. Accordingly, implementation of comprehensive real-time monitoring systems including subsequent data processing in fresh fruit and vegetable supply chains is still rare. Only few studies apply an extensive system in practice [97,103,106,117]. Establishing a system that is genuinely scalable and easily transferable to other fruit and vegetables or supply chains remains an unsolved challenge. Until now, solutions have to be customized to each supply chain, because generic concepts for implementation are missing. This includes guidelines on the choice of technology and knowledge of process-related, technical and actor-related requirements, as discussed above. For exploitation of the full potential of IoT-based ambient parameter monitoring systems, these knowledge gaps need to be filled. 

Large scale implementation of IoT-based monitoring could also facilitate the application of other quality-related technologies. Solutions are in development for directly measuring product quality parameters. These include physical and chemical characteristics such as the soluble solids content, dry matter or firmness [126]. Emerging technologies in this field are near-infrared (NIR) spectroscopy, multi- and hyperspectral imaging, freshness sensors and E-noses which have already been studied for various products [126,127,128,129,130]. Direct measurement of quality parameters at specific processing steps can help in determining the optimum harvest time, in sorting of products to more uniform batches and in supplying additional information on ripening status and shelf life [129,130]. Possibly, these technologies could also routinely be used after an ambient parameter monitoring system noticed a deviation, e.g., a temperature abuse, for determining certain quality parameters and making logistic decisions. Further research on integration of these technologies with an ambient parameter monitoring system could help to explore synergies and benefits regarding quality retention and food loss prevention. 

## 6. Conclusions

The ongoing progress in the field of IoT creates new possibilities in the fresh fruit and vegetable sector. Suitable sensor and communication technologies for real-time monitoring of ambient parameters relevant for quality and shelf life are available or in development. Although fresh fruit and vegetables have various quality characteristics, many possible applications for adding value to supply chains were identified. For managerial decision-making, the improved transparency about the quality status of products can be used for optimized logistic control. Decisions can be based on a solid data basis and instead of rejecting batches with suboptimal quality, alternative distribution strategies or channels can be considered. From a dietary perspective, a real-time monitoring system enables products with high nutritional value, because the ambient conditions during storage and transport are directly connected to the preservation of nutrients. Additionally, consistently high product qualities at the point of sale can benefit food marketing. Several studies show the promising potential of applications for optimized product qualities and reductions in products lost and wasted along the supply chain. However, there is still a lack of studies implementing a comprehensive monitoring system including device, network and application layers in a real scenario. The complexity and heterogeneity of fresh produce and the supply chains as well as technical challenges hinder implementation. Until now, there is no “one-size-fits-all” solution. Therefore, further knowledge on supply chain-specific and process-related factors is needed, as well as generic concepts for implementing an IoT-enabled monitoring system for fresh fruit and vegetables.

## Figures and Tables

**Table 1:** Overview of ambient parameters and corresponding sensor technology for an IoT application.

Ambient Parameter	Sensor Technology Suitable for IoT	Solutions Commercially Available	Examples	Relevant Fruit and Vegetables	References
Temperature	Thermistor Resistive temperature device	Yes	Sentrius^TM^ RS1xx by Laird^TM^ Connectivity (LoRa) Sentrius^TM^ BT510 Laird^TM^ Connectivity (Bluetooth) Piccolo TMX by WirelessLinks (cellular communication)Wireless temperature and humidity logger by Efento (NB-IoT) LAS-603V2 T/RH Sensor by kiwi technology Inc. (LoRa) XSense^®^RT-2G by XSense^®^ (cellular communication) freshtime by infratab (RFID/NFC)	All commodities, particularly for: berry fruit, leafy vegetables, fresh herbs, pineapples, papayas, mangoes, avocadoes, bananas	[6,46,61,62,63,64,65,66,67,21]
Relative Humidity	Resistive hygrometer Capacitive hygrometer	Yes	Sentrius RS1xx by Laird^TM^ Connectivity (LoRa) Wireless temperature and humidity logger by Efento (NB-IoT) LAS-603V2 T/RH Sensor by kiwi technology Inc. (LoRa) DRA LHT65 by Dragino (LoRaWAN) TempTale Geo LTE by Sensitech (cellular communication) Pod Humidity PA3 by Verigo (Bluetooth) XSense^®^RT-2G by XSense^®^ (cellular communication)	All commodities, particularly for: leafy vegetables, fruit-vegetables, fresh herbs, berry fruit, citrus fruit In long-term storage: pumpkins, onions, garlic	[6,21,49,61,64,65,66,68,69,70]
Ethylene	Chemiresistors Chemicapacitors	No (only sensing units without battery or wireless connectivity)	Sensing units: Membrapor Ethene, Ethylene Gas Sensor C_2_H_4_/C-10 by Membrapor Electrochemical Ethylene Gas Sensor ME3-C_2_H_4_ by Winsen	Climacteric fruit, particularly for bananas, leafy vegetables, broccoli, cauliflower, cucumbers, carrots, eggplant, citrus fruit, watermelons, bell pepper, pumpkin, green beans, pears, blackberries, raspberries,	[6,21,50,71,72]
Oxygen	Electrochemical sensor Fluorescence sensor	Yes	ECgaspoint by EC Sense (Wi-Fi) PS-3217 by Pasco (Bluetooth)	During CA storage/transport: apples, pears, cabbages, onions, kiwifruit, avocados, melons, nectarines, peaches, broccoli, bananas, cherries, figs, kakis, asparagus, mangoes, plums, strawberriers	[6,21,73,74]
Carbon Dioxide	NDIR sensor Metal oxide sensor	Yes	PS-3208 by Pasco (Bluetooth) SenseCAP Wireless CO_2_ Sensor by Seeed Studio (LoRa)	During CA storage/transport: same as for oxygen Climacteric fruit	[6,75,76,21]
Shock/ vibration	MEMS accelerometer	Yes	Sentrius^TM^ BT510 Laird^TM^ Connectivity (Bluetooth) BeanDevice^®^WIFI|Wilow^®^ AX-3D by BeanAir (Wi-Fi) BeanDevice^®^WIFI|Wilow^®^ AX-3DS by BeanAir (Wi-Fi)	All commodities, particularly for: berry fruit, pineapples, cherries, tomatoes, mushrooms, courgettes, bananas	[21,59,60,62,77]

**Table 2:** Overview of advantages, disadvantages and example applications of wireless communication technologies in the context of parameter monitoring in fresh fruit and vegetable supply chains.

Communication Technology	Characteristics	Studies Including Examples of Applications	References
**NFC** Costs Power consumption Transmission range Frequency Standardization Additional aspects	Low Low Very short (up to 10 cm) Unlicensed, 13.56 MHz Standard ECMA- 340, ISO/IEC 18092 Easy integration (smartphone readable)	Tracking of temperature information at different process steps (test was carried out in a meat supply chain, but concept is applicable to other products)	[82,98,99]
**RFID** Costs Power consumption Transmission range Frequency Standardization Additional aspects	Low (passive/semi-passive tags) High (active tags) Low (passive/semi-passive tags)High (active tags) Short (up to 100 m) Unlicensed, 125–148 KHz (LF); 13.56 MHz (HF), 433 MHz, 866–930 MHz (UHF), 2.45 GHz, 5.8 GHz (Microwave) Lack of uniform global standards Readability problems for UHF (e.g., metal, high water environment)	Monitoring of temperature, RH, vibration, O_2_, CO_2_, ethylene in fresh garlic scape supply chain (in combination with cellular technology) Monitoring of temperature and RH in commercial fruit storage warehouses (in combination with ZigBee) Monitoring of temperature, RH, O_2_, CO_2_, vibration/shock in sweet melon supply chain (in combination with cellular technology or Wi-Fi) Monitoring of temperature in kiwifruit supply chain (in combination with cellular technology)	[10,85,96,100,101,102,13,96,97,103]
**ZigBee** Costs Power consumption Transmission range Frequency Standardization	Low Low Short (up to 100 m) Unlicensed, 868/915 MHz; 2.4 GHz Standard 802.15.4	Monitoring of temperature and RH in commercial fruit storage warehouses (in combination with RFID) Monitoring of temperature, RH and vibration during truck transport of lettuce	[13,81,83,90,95]
**Bluetooth** Costs Power consumption Transmission range Frequency Standardization Additional aspects	Low Low Short (10–100 m) Unlicensed, 2.4 GHz Standard 802.15.1 Easy integration (smartphone readable)	Monitoring of temperature, RH and CO_2_ concentration in storage of oranges (in combination with Wi-Fi) Monitoring of ascorbic acid content in fresh parsley	[81,82,83,104,105]
**Wi-Fi** Costs Power consumption Transmission range Frequency Standardization Additional aspects	Low High (if sensors are connected directly to Wi-Fi) Short (up to 100 m) Unlicensed, 2.4 GHz Standard IEEE 802.11 Easy integration (smartphone compatible)	Monitoring of temperature, RH, O_2_, CO_2_, vibration/shock in sweet melon supply chain (in combination with RFID) Monitoring of temperature during land transportation of iceberg lettuce	[81,83,96,106]
**Cellular communication** Costs Power consumption Transmission range Frequency Standardization Additional aspects	High High (if sensors are connected directly to cellular communication) Long (several km) Licensed, 1.7–2.7 GHz (2G/3G4G); 3.4–3.6 GHz (5G) Different standards, depending on technology Large coverage Easy integration (smartphone compatible)	Monitoring of temperature, RH, vibration, O_2_, CO_2_, ethylene in fresh garlic scape supply chain (in combination with RFID) Monitoring of temperature, RH, O_2_, CO_2_, vibration/shock in sweet melon supply chain (in combination with RFID) Monitoring of temperature in kiwifruit supply chain (in combination with RFID) Monitoring of temperature, RH and CO_2_ in a simulated peaches and nectarine supply chain (in combination with RFID)	[80,81,83,96,97,103,107,108,109]
**LPWAN** Costs Power consumption Transmission range Frequency Standardization Additional aspects	Low (operation in unlicensed spectrum) High (operation in licensed spectrum) Low Long (several km) Unlicensed and Licensed, depending on technology Different standards, depending on technology Coverage in suburban and rural areas might be incomplete	Monitoring of temperature RH in tomato greenhouses (LoRa in combination with Wi-Fi) Monitoring of temperature, RH and CO_2_ concentration in tomato greenhouse (LoRa)	[80,81,90,107,110,111]

## Data Availability

Not applicable.
